# Parental Transmission of Type 2 Diabetes Risk in Offspring: A Prospective Family-Based Cohort Study in Northern China

**DOI:** 10.3390/nu17081361

**Published:** 2025-04-16

**Authors:** Hexiang Peng, Mengying Wang, Huangda Guo, Tianjiao Hou, Yixin Li, Hanyu Zhang, Yinxi Tan, Xueying Qin, Yiqun Wu, Dafang Chen, Jing Li, Yonghua Hu, Tao Wu

**Affiliations:** 1 Department of Epidemiology and Biostatistics, School of Public Health, Peking University, Beijing 100191, China; 2 Key Laboratory of Epidemiology of Major Diseases (Peking University), Ministry of Education, Beijing 100191, China; 3 Department of Nutrition and Food Hygiene, School of Public Health, Peking University, Beijing 100191, China

**Keywords:** type 2 diabetes, maternal, paternal, offspring, family aggregation, parent of origin effect

## Abstract

**Background:** While parental type 2 diabetes (T2D) is a known risk factor for offspring T2D, the differential impact of maternal versus paternal transmission remains debated. **Methods:** This prospective family-based cohort study enrolled 4508 diabetes-free adults from Northern China with a median 7.32-year follow-up. Using Cox proportional hazards models, we examined parent-of-origin effects on T2D incidence, adjusting for lifestyle, adiposity, and metabolic covariates. **Results:** Parental T2D conferred elevated offspring risk (adjusted HR = 1.82, 95% CI:1.44–2.30), and was predominantly driven by maternal transmission. Maternal T2D was robustly associated with offspring risk (HR = 1.89, 95% CI: 1.47–2.43), whereas paternal T2D showed no significant effect (HR = 1.27, 95% CI: 0.88–1.84). Offspring with only maternal T2D history exhibited the highest risk (HR = 2.55, 95% CI: 1.87–3.50; *p* = 4.70 × 10^−9^), persisting after full adjustment, while no significant association was observed for paternal diabetes. Lifestyle modified this association: healthy diet (diet score > 2 vs. ≤2: HR = 1.34 vs. 2.76; *p*_interaction_ = 9.10 × 10^−4^) and regular exercise (regular vs. unregular: HR = 1.13 vs. 2.10; *p*_interaction_ = 4.20 × 10^−2^) attenuated maternal transmission. **Conclusions:** Maternal T2D confers greater intergenerational risk than paternal T2D, with modifiable lifestyle factors mitigating this association. These findings highlight the importance of integrating maternal diabetes history into clinical risk stratification tools and prioritizing lifestyle interventions in the offspring of affected mothers to mitigate inherited risk.

## 1. Introduction

Type 2 diabetes (T2D) has become a significant global public health challenge, accounting for 90% of diabetes. In 2021, the World Health Organization (WHO) reported that there were 529 million people diagnosed with diabetes worldwide, a number expected to rise to 1.31 billion by 2050 [1]. China has the highest number of diabetes patients globally, with over 118 million individuals living with the disease, representing approximately 22% of the global diabetes population [2]. This underscores the urgent need to investigate the etiology of T2D and develop effective prevention and control strategies. T2D is a complex disease influenced by both genetic and environmental factors, with genetic factors contributing to up to 69% of the risk in individuals aged 35 to 60 [3]. Despite numerous genetic variants associated with T2D being identified through genome-wide association studies, these variants explain only a small fraction (approximately 10%) of the heritability, posing a major challenge in addressing the missing heritability and further exploring the disease’s genetic underpinnings [4].

Family history of T2D is a well-established risk factor reflecting both genetic and environmental influences. Previous studies have shown that individuals with a family history of T2D (particularly first-degree relatives, such as parents or siblings) are at higher risk of developing the disease, often at an earlier age [5]. Notably, female offspring with a maternal history of T2D face a greater risk of developing the disease, whereas this risk is not observed in males [6].

However, most studies have broadly examined the impact of having a first-degree relative with the disease or the number of affected parents on the offspring’s risk of developing T2D, without differentiating whether the risk varies between paternal and maternal inheritance. A case–control study demonstrated that children of mothers with T2D have a higher risk of developing T2D compared to children of fathers with T2D [7]. However, the sample size was small, and case–control studies are limited in their ability to establish causal relationships, making them less robust than cohort studies in terms of evidence strength. And a cohort study demonstrated that a parental history of type 2 diabetes (T2D) may be a stronger predictor of longitudinal glycemic trends than race/ethnicity [8]. Moreover, another study investigated the impact of paternal diabetes on pre-diabetic phenotypes in adult offspring [9]. It found that offspring of fathers with early-onset diabetes and non-diabetic mothers were leaner and exhibited lower early insulin secretion, suggesting a paternal imprinting effect [9].

A previous study examined the impact of maternal diabetes on cardiometabolic risk in offspring and found that a maternal history of diabetes was associated with both insulin resistance and impaired first-phase insulin secretion, suggesting a potential mechanism for transgenerational transmission of diabetes [10]. However, few studies have quantitatively assessed the impact of parental diabetes on offspring’s type 2 diabetes and related indicators, and most of these studies have small sample sizes and a cross-sectional design. More robust evidence is still needed to provide insights into the parent origin effects of diabetes. A more detailed exploration of the effect of parental diabetes origin on offspring could provide better insights into the etiology of type 2 diabetes, and may also help partially address the issue of missing heritability.

Therefore, this prospective family-based cohort study aimed to elucidate parental transmission patterns of T2D risk in Chinese adult offspring, leveraging the large-scale Beijing Fangshan family-based cohort to address critical knowledge gaps in understanding parental diabetes transmission in Asian populations.

## 2. Methods

### 2.1. Population

The study participants were derived from the Beijing Fangshan family-based cohort. The cohort utilized a “proband recruitment” method, where probands were recruited through chronic disease management channels in the community, and their parents and siblings were subsequently invited to participate in the survey. Baseline investigations for the cohort began in June 2005 and were primarily completed between 2013 and 2017. Participants were followed-up over the long term to collect the risk factors related to chronic disease outcomes, aiming to investigate the genetic and environmental risk factors associated with the disease. The first follow-up period was conducted between July 2019 and December 2020, with an average follow-up duration of six years for the cohort. Recruitment, data collection, and biological sample collection were carried out through the community health service system. Detailed descriptions of the cohort have been provided in previously published articles [11,12].

In the current study, a total of 4508 participants who were free of type 1 and type 2 diabetes at baseline were included. The study was approved by the Institutional Review Board of Peking University Health Science Center (IRB00001052-13027). Informed consent was obtained from all participants.

### 2.2. Exposure and Outcome

The primary exposure was self-reported parental T2D status (yes/no) for both father and mother from the questionnaire. Each study subject was asked whether their parents have been diagnosed with diabetes, with the following response options: “yes”, “no”, “don’t know”. Self-reported parental diabetes was consistent with true parental diabetes status when both parents were in the cohort. Based on this information, participants were categorized into four groups: neither parent with T2D, mother with T2D but father without, father with T2D but mother without, and both parents with T2D. As for the T2D definition for offspring, individuals were classified as having T2D if they met any of the following criteria, while excluding other specific types of diabetes: (a) a documented diagnosis of T2D (International Statistical Classification of Diseases and Related Health Problems, Tenth Revision [ICD-10], code E11); (b) HbA1c ≥ 6.5%; (c) fasting blood glucose (FBG) ≥ 7.0 mmol/L; (d) use of glucose-lowering medications; or (e) exclusion of other specific types of diabetes.

### 2.3. Measurements

Trained investigators collected information from all participants during the baseline survey using a qualified questionnaire. Data collected included general sociodemographic characteristics (age, sex), lifestyle factors (smoking, drinking, physical activity, diet, sleep duration), medical history, family history, and medication use. Standardized equipment was used to measure height and weight. Body mass index (BMI) was calculated using the following formula: BMI = weight (kg)/height (m)^2^. Daily consumption of fish, processed meat, fruits, and vegetables (yes/no) were used to construct a dietary score, ranging from 0 to 4. Each healthy diet habit was assigned 1 point, resulting in a total score ranging from 0 to 4. A diet score of ≤2 was classified as an unhealthy diet, while a score of >2 was considered indicative of a healthy diet.

Biochemical indicators were assessed using fasting blood samples obtained from participants. Trained investigators collected venous blood samples in the morning after participants fasted for at least 8 h before blood collection. Samples were immediately centrifuged, and plasma and serum were stored at −80 °C until fasting blood glucose, glycated hemoglobin (HbA1c), and blood lipid-related indicators were measured. All procedures adhered to standardized protocols to ensure consistency and reliability.

### 2.4. Statistical Analysis

Clinical characteristics were descriptively compared between offspring with none, mother, father, or 2 parents with T2D. Chi-square tests and analysis of variance were used to examine the difference among different groups. The continuous variables were presented as means and standard deviations, and categorical variables were expressed as numbers and percentages. The family-based multilevel Cox regression analysis was conducted using the survival and coxme packages in R (version 4.3.1). The coxme function enables the classic Cox proportional hazards model to include random effects (e.g., familial or group effects) to account for correlations within family structures. This makes it particularly suitable for handling data with familial or clustered structures. Model 1 was adjusted for age (continuous), sex (female, male), and BMI (continuous). Model 2 was further adjusted for smoking status (never smoked, current smoker), alcohol consumption (never drank, current drinker), systolic blood pressure (continuous), and sleep duration (continuous) based on Model 1. Model 3 further adjusted for dietary factors (diet score ≤ 2, diet score > 2) and regular exercise (regular, unregular) based on model 2.

In our study, stratification variables were selected based on their known association with the outcome variable and their potential to introduce confounding. Stratification analysis was explored by grouping participants based on age, sex, BMI, smoking, alcohol consumption, sleep duration, diet group, and exercise. Regarding sensitivity models, we performed analyses to assess the robustness of our findings under different assumptions and to address potential biases. These analyses further adjusted for fasting blood glucose levels (continuous), use of antihypertensive, antidiabetic, and lipid-lowering drugs, as well as history of cardiovascular disease (including stroke and hypertension). This comprehensive approach ensures that our results are reliable and valid, providing a more complete picture of the relationship under investigation.

## 3. Results

### 3.1. Baseline Characteristics of Study Population

The baseline characteristics of the study sample stratified by parental T2D status are displayed in Table 1. A total of 4508 individuals were included in the longitudinal study, comprising 2334 males (51.8%) and 2174 females (48.2%). The median follow-up duration was 7.32 years, during which 808 new cases of T2D (17.9%) were identified. The average age of participants was 56.4 years (SD = 11.3). Among the participants, 3845 (85.3%) had no parental history of diabetes, 163 (3.6%) had only paternal diabetes history, 437 (9.7%) had only maternal diabetes history, and 63 (1.4%) had a history of both parents with diabetes.

Compared with those who did not develop incident T2D, individuals with incident T2D tended to have higher BMI, longer sleep duration, higher fasting blood glucose, and higher systolic blood pressure, as well as a higher prevalence of hypertension (all *p* < 0.001). No intergroup differences emerged in age, sex, smoking, drinking, exercise, or history of stroke (Table 1). Notably, maternal T2D prevalence differed significantly between T2D incident (14.4%) and non-incident groups (10.4%), whereas paternal T2D prevalence remained comparable (4.8% vs. 5.8%; *p* = 0.21). These findings suggest maternal-specific transmission patterns independent of conventional risk factors, warranting further investigation through multivariable-adjusted models.

### 3.2. Association of Parental T2D with Offspring Risk of T2D

**Figure 1:**
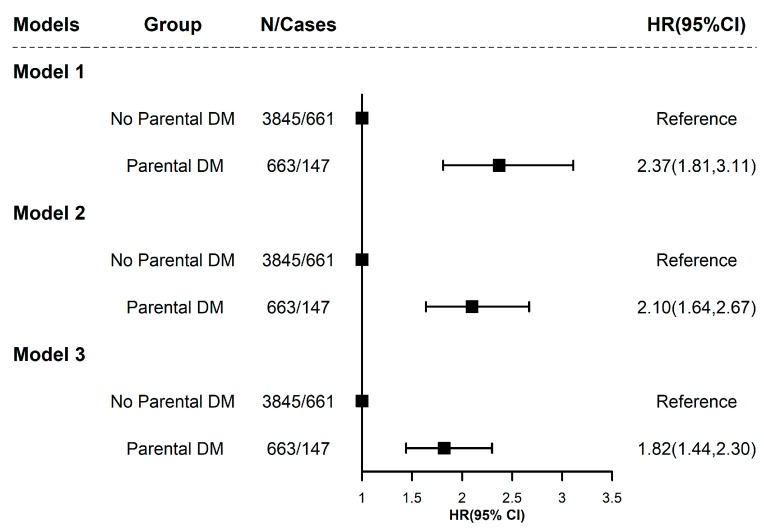
Association between parental history of diabetes and offspring risk of type 2 diabetes. Model 1 adjusted for age (continuous variable), sex (female, male), and BMI (continuous variable). Model 2 further adjusted for smoking status (never smoked, current smoker), alcohol consumption (never drank, current drinker), systolic blood pressure (continuous variable), and sleep duration (continuous variable) based on Model 1. Model 3 further adjusted for dietary factors (diet score ≤ 2, diet score > 2) and regular exercise (regular, irregular) based on Model 2.

As shown in Figure 1, sequential multivariable-adjusted Cox regression analyses revealed a robust association between parental diabetes history and offspring T2D risk. In the baseline model adjusted for age, sex, and BMI (Model 1), individuals with parental diabetes exhibited a 2.37-fold increased risk compared to those without parental history (HR = 2.37, 95% CI: 1.81–3.11, *p* = 5.10 × 10^−10^). Partial attenuation occurred after additional adjustment for smoking, alcohol use, systolic blood pressure, and sleep duration (Model 2: HR = 2.10, 95% CI: 1.64–2.67, *p* = 2.70 × 10^−9^), with further mitigation in the fully adjusted model incorporating dietary patterns and exercise frequency (Model 3: HR = 1.82, 95% CI: 1.44–2.30, *p* = 4.60 × 10^−7^). The persistence of significant risk elevation across progressive adjustments underscores parental diabetes as an independent predictor of offspring diabetes development, which emphasizes the clinical importance of integrating parental diabetes status into risk assessment frameworks.

### 3.3. Maternal vs. Paternal History of Diabetes

Table 2 presents the results of the association between paternal and maternal history of diabetes and offspring risk of T2D. Maternal diabetes demonstrated persistent associations across adjustment models, with fully adjusted hazard ratios remaining statistically significant (Model 3: HR = 1.89, 95% CI: 1.47–2.43), whereas paternal associations were attenuated to non-significance after full adjustment (Model 3: HR = 1.27, 95% CI: 0.88–1.84) after further adjustments.

When combining maternal and paternal diabetes histories, offspring exposed to maternal T2D alone exhibited substantially elevated risk after full adjustment (Model 3: HR = 1.95, 95% CI: 1.50–2.55). In contrast, paternal associations lose significance after full adjustment (Model 3: HR = 1.39, 95% CI: 0.89–2.17), reinforcing maternal predominance. Furthermore, biparental diabetes history was strongly associated with offspring T2D risk in all models (HR = 2.07, 95% CI: 1.12–3.83). These findings underscore maternal T2D as a dominant intergenerational risk factor, with biparental history identifying a high-risk subgroup warranting intensified preventive interventions.

### 3.4. Results of Stratification Analysis

Stratified multivariable analyses using the fully adjusted model revealed distinct parental transmission patterns (Table 3). Paternal T2D showed no significant association with offspring risk across all subgroups (*p* > 0.05). Conversely, maternal T2D demonstrated persistently significant associations with offspring T2D risk in nearly all strata (*p* < 0.05), confirming maternal-specific transmission robustness. Overall, the results were robust. Notably, significant effect modification by diet score and exercise was observed, with *p*-values for interaction of 9.10 × 10^−4^ and 4.20 × 10^−2^, respectively. Offspring with a healthy diet score and regular exercise were found to have a lower risk of T2D, even when their mother had a history of diabetes. These findings underscore modifiable lifestyle factors as critical buffers against maternally transmitted diabetes risk, highlighting targeted prevention opportunities for high-risk offspring through behavioral interventions.

### 3.5. Results of Sensitivity Analysis

To assess result robustness, we conducted additional analyses incorporating fasting blood glucose (continuous), medication use (categorical), and cardiovascular disease history (binary) into the fully adjusted model. The results remained consistent with the primary findings (Table 4), indicating that only maternal T2D was strongly and significantly associated with an increased risk of T2D in offspring, while paternal T2D showed no such association. This consistency across model specifications strengthens evidence for distinct parental transmission mechanisms, suggesting maternal T2D influences offspring risk through pathways less confounded by cardiometabolic comorbidities. The null association with paternal diabetes further supports the biological plausibility of parent-of-origin disease transmission independent of shared environmental factors.

## 4. Discussion

To our knowledge, this is the first cohort study to investigate the association between parental T2D and the risk of T2D in offspring. We found that a parental history of diabetes was associated with a higher risk of T2D in offspring, with this association being particularly significant for maternal history of T2D. Specifically, maternal diabetes was strongly linked to an increased risk of T2D in offspring, while no such association was observed for paternal diabetes. These results remained robust in both stratification and sensitivity analyses. Additionally, the association between maternal diabetes and offspring risk of T2D may be modified by dietary factors and exercise. These findings suggest that maternal diabetes consistently has a more substantial impact on offspring’s risk of developing T2D than paternal diabetes, highlighting the potential role of parent-of-origin effects in the development of T2D.

Our study employed a prospective design to quantify the impact of parental diabetes on the risk of type 2 diabetes (T2D) in offspring, suggesting that T2D is a complex disease influenced by genetic factors. Previous twin or familial studies have reported a heritability estimate for diabetes ranging from 20% to 80% [13,14], indicating a moderate to high genetic influence on T2D. Family history is a comprehensive indicator of type 2 diabetes determinants [15], reflecting both genetic factors and environmental influences such as obesity, diet, and socioeconomic status, and it can span multiple generations [16,17,18]. Parental transmission plays a major role in the high aggregation of T2D in Iranian families [19]. The risk of diabetes in offspring is increased by two to six times when parents have diabetes, with the highest risk observed when both parents are affected [14,20,21]. The EPIC-InterAct study found that a family history of T2D was associated with a higher incidence (HR 2.72, 95% CI: 2.48–2.99), with the greatest risk observed in individuals with a biparental history (HR 5.14, 95% CI: 3.74–7.07) or when parents were diagnosed before age 50 (HR 4.69, 95% CI: 3.35–6.58), particularly for those with a maternal family history [22]. However, they found that the genetic score explained only 2% of the family history-associated risk of T2D, emphasizing that family history remains a strong, independent, and easily assessable risk factor for the disease [22]. Evidence from a U.S. study also suggests that the diabetes prevalence for individuals with a family history was more than four times higher than the prevalence for individuals without a family history [23]. The relationship of family history and risk of T2D differs by ancestry [24,25]. The current cohort was based on family-based populations from north China and used a proband approach to select participants. In contrast, previous studies were based on general population data, predominantly from European and American cohorts.

The risk of T2D is higher in offspring if the mother rather than the father has T2D [26], but the conclusion did not reach consistency. The National Health and Nutrition Examination Survey demonstrated that the prevalence for individuals with a maternal diabetes history is higher than those with a paternal diabetes history (16.5% vs. 12.4%), which is consistent with our study [23]. A previous study highlighted the high proportion of maternal T2D and suggested the role of adenosine deaminase and phosphoglucomutase in maternal transmission of T2D [27]. A Dutch analysis found that after adjusting for diet, lifestyle factors, and adiposity, the hazard ratios for diabetes transmission from maternal (HR = 2.20; 95% CI: 1.87–2.60) and paternal (HR = 2.23; 95% CI: 1.77–2.80) history were similar [28]. Meanwhile, the Framingham Offspring Study found that both maternal and paternal diabetes conferred similar risks for offspring T2D, but offspring with maternal diabetes were slightly more likely to have abnormal glucose tolerance compared to those with paternal diabetes [14]. In our study, we observed a stronger association between maternal history of T2D and offspring risk of developing T2D, which is consistent with the previous studies. This difference may be partly attributed to genetic and environmental factors, including X-linked traits, mitochondrial inheritance, gene imprinting, intrauterine programming, and the more prominent maternal role in raising children [21,26,29]. These factors may contribute to partly explain the observed maternal effect in offspring T2D risk.

A family history of diabetes can partially represent the complex effects of genetic and environmental factors on offspring. Prominent lifestyle and anthropometric and genetic risk factors explained only a marginal proportion of the family history-associated excess risk [22]. However, the mechanisms behind the differential impacts of parents on their children may be quite complex. Genomics imprinting refers to the phenomenon where alleles from both parents are expressed in a manner that favors the expression of one parent’s allele, while the other parent’s allele is either not expressed or partially expressed. Specifically, this may manifest as differences in the risk to offspring depending on whether the father or mother has the condition. Emerging evidence for parent-of-origin effects at T2D susceptibility loci has been summarized [26], highlighting the need for dedicated genetic studies to determine whether risk alleles exhibit differential effects based on their maternal or paternal transmission. However, the addition of genetic risk scores of up to 20 T2D-associated variants improved little in explaining the link between family history and T2D risk [30,31]. In addition to genetic factors, the intergenerational transmission of disease risk from the maternal line may also be influenced by various non-genetic mechanisms, including the persistence of maternal exposure to external stressors, indirect effects related to parental physiology, and epigenetic mechanisms [16]. Moreover, gene–environment interaction might also partly explain this phenomenon. We found that the risk of maternal diabetes in offspring might be modified by healthy diet and regular exercise, which was consistent with the previous study, in which maternal transmission of risk of diabetes was explained by diet (9.4%), physical activity, and by adiposity, i.e., body mass index and waist and hip circumference (23.5%) [28]. Additionally, human studies have shown that impaired insulin secretion contributes to the abnormal glucose tolerance observed in adult offspring exposed to maternal diabetes, with insulin secretion potentially being reduced even in offspring with normal glucose tolerance [16].

This study has several strengths. First, this study represents the first comprehensive investigation of the association between parental T2D and offspring T2D, while examining the extent of this impact and distinguishing between maternal and paternal effects. These findings could provide valuable clues for future parent-offspring studies, such as uncovering allele-specific effects inherited from mothers or fathers at the genetic level, thereby offering more detailed insights into the precise prevention and management of T2D. Second, this study leverages the Beijing family cohort, which includes comprehensive family history data. Notably, the variable indicating parental T2D status was fully reported without any missing data in the questionnaire. However, this study also has several limitations. The self-reported parental T2D status may introduce misclassification bias. Possible over-representation of maternal transmission of diabetes might be observed in population studies and subjects may be more likely to know health status of their mothers than of their fathers [32,33]. Moreover, the study provides only preliminary evidence of a parental origin effect on T2D at the phenotypic level, requiring further validation through genetic studies, such as exploring differential allele expression inherited from parents using genomic data. Additionally, residual confounding cannot be ruled out, as some confounding factors may not have been fully accounted for, potentially leading to bias. A previous study demonstrated the age onset of familial diabetes and found that individuals with a family member (parents or sibling) diagnosed with diabetes at a younger age are more likely to develop diabetes themselves, and are also at a higher risk of developing it at an earlier age compared to those with a family member diagnosed in later life [34]. Our analysis did not account for parental age of diabetes onset, nor parental metabolic characteristics during offspring development. Future work should integrate genomic data (e.g., allele-specific expression analysis) with longitudinal parental health metrics to disentangle genetic, epigenetic, and environmental contributions. These refinements could enable risk stratification based on parental transmission patterns and developmental critical windows, advancing personalized prevention paradigms. While our findings provide mechanistic insights into parent-of-origin effects, the convenience sample origin of the data may limit generalizability to populations with distinct genetic ancestry or socioeconomic contexts. However, the consistency of our POE effects with prior studies suggests biological robustness. Future replication in population-based cohorts with diverse ancestry is warranted.

## 5. Conclusions

Our findings demonstrate a pronounced maternal predominance in T2D transmission, suggesting that T2D may be influenced by parent-of-origin effects. Importantly, this transmission pattern appears modifiable through lifestyle interventions, with dietary optimization and regular exercise attenuating maternal transmission risk. These results advocate clinical implementation of parent-specific risk stratification protocols, particularly recommending intensive lifestyle counseling for offspring of diabetic mothers during critical windows like preconception and early adulthood. To further deepen our understanding, future studies utilizing genomic data are needed to identify T2D-related genetic variants associated with parent-of-origin effects and to uncover the underlying mechanisms.

## Figures and Tables

**Table 1:** Characteristics of participants stratified by incident type 2 diabetes status.

Characteristics		Overall	Incident T2D		*p*
			No	Yes	
Number		4508	3700	808	
Sex (%)	Female	2174 (48.2)	1790 (48.4)	384 (47.5)	0.688
	Male	2334 (51.8)	1910 (51.6)	424 (52.5)	
Age (mean (SD))		56.4 (11.3)	56.3 (11.6)	56.7 (9.7)	0.362
BMI (mean (SD))		25.9 (3.6)	25.7 (3.6)	26.6 (3.6)	<0.001
Smoking (%)	Never	2361 (52.4)	1938 (52.4)	423 (52.4)	1
	Ever/current	2147 (47.6)	1762 (47.6)	385 (47.6)	
Drinking (%)	Never	2800 (62.1)	2294 (62.0)	506 (62.6)	0.771
	Ever/current	1708 (37.9)	1406 (38.0)	302 (37.4)	
Sleep hour (mean (SD))		7.07 (2.37)	6.98 (2.49)	7.48 (1.72)	<0.001
Diet score (%)	≤2	2166 (48.0)	1838 (49.7)	328 (40.6)	<0.001
	>2	2342 (52.0)	1862 (50.3)	480 (59.4)	
Exercise (%)	Unregular	4034 (89.5)	3314 (89.6)	720 (89.1)	0.748
	Regular	474 (10.5)	386 (10.4)	88 (10.9)	
Fasting glucose (mean (SD))		4.8 (0.9)	4.8 (0.9)	4.9 (1.0)	0.006
SBP (mean (SD))		137.3 (20.7)	137.0 (20.9)	138.8 (20.1)	0.019
DBP (mean (SD))		82.7 (14.7)	82.6 (15.2)	82.9 (11.9)	0.56
Antihypertension drugs (%)	No	2686 (59.6)	2311 (62.5)	375 (46.4)	<0.001
	Yes	1822 (40.4)	1389 (37.5)	433 (53.6)	
Antidiabetic drugs (%)	No	4508 (100.0)	3700 (100.0)	808 (100.0)	NA
Antihyperlipidemic drugs (%)	No	4087 (90.7)	3394 (91.7)	693 (85.8)	<0.001
	Yes	421 (9.3)	306 (8.3)	115 (14.2)	
Hypertension (%)	No	1521 (33.7)	1303 (35.2)	218 (27.0)	<0.001
	Yes	2987 (66.3)	2397 (64.8)	590 (73.0)	
Stroke (%)	No	2914 (64.6)	2412 (65.2)	502 (62.1)	0.108
	Yes	1594 (35.4)	1288 (34.8)	306 (37.9)	
Paternal diabetes (%)	No	4282 (95.0)	3521 (95.2)	761 (94.2)	0.286
	Yes	226 (5.0)	179 (4.8)	47 (5.8)	
Maternal diabetes (%)	No	4008 (88.9)	3316 (89.6)	692 (85.6)	0.001
	Yes	500 (11.1)	384 (10.4)	116 (14.4)	
Parental diabetes (%)	No parents	3845 (85.3)	3184 (86.1)	661 (81.8)	0.011
	Only maternal	437 (9.7)	337 (9.1)	100 (12.4)	
	Only paternal	163 (3.6)	132 (3.6)	31 (3.8)	
	Both parents	63 (1.4)	47 (1.3)	16 (2.0)	

BMI, body mass index; SBP, systolic blood pressure; DBP, diastolic blood pressure.

**Table 2:** Association between parental diabetes history and offspring risk of type 2 diabetes.

Variables		N/Cases	Model 1		Model 2		Model 3	
			HR (95% CI)	*p*	HR (95% CI)	*p*	HR (95% CI)	*p*
Paternal diabetes								
	No	4282/761	1.00		1.00		1.00	
	Yes	226/47	1.54 (0.99, 2.39)	5.40 × 10^−2^	1.36 (0.92, 2.01)	1.50 × 10^−1^	1.27 (0.88, 1.84)	2.10 × 10^−1^
Maternal diabetes								
	No	3508/692	1.00		1.00		1.00	
	Yes	500/116	2.38 (1.77, 3.21)	1.20 × 10^−8^	2.15 (1.65, 2.80)	1.50 × 10^−8^	1.89 (1.47, 2.43)	7.00 × 10^−7^
Combined								
	No parents	3845/661	1.00		1.00		1.00	
	Only maternal	437/100	2.55 (1.87, 3.50)	4.70 × 10^−9^	2.27 (1.72, 3.00)	8.40 × 10^−9^	1.95 (1.50, 2.55)	8.80 × 10^−7^
	Only paternal	163/31	1.86 (1.12, 3.10)	1.70 × 10^−2^	1.60 (1.00, 2.54)	4.70 × 10^−2^	1.39 (0.89, 2.17)	1.50 × 10^−1^
	Both parents	63/16	2.54 (1.18, 5.51)	1.80 × 10^−2^	2.23 (1.15, 4.32)	1.70 × 10^−2^	2.07 (1.12, 3.83)	2.10 × 10^−2^

Model 1 adjusted for age (continuous variable), sex (female, male), and BMI (continuous variable). Model 2 further adjusted for smoking status (never smoked, current smoker), alcohol consumption (never drank, current drinker), systolic blood pressure (continuous variable), and sleep duration (continuous variable) based on Model 1. Model 3 further adjusted for dietary factors (diet score ≤ 2, diet score > 2) and regular exercise (regular, irregular) based on Model 2. For single factor analysis, paternal diabetes and maternal diabetes were adjusted, respectively.

**Table 3:** Subgroup analysis for the association between parental history of diabetes and offspring risk of type 2 diabetes.

Stratified Factors		Paternal Diabetes			Maternal Diabetes		
		N/Cases	No	Yes	N/Cases	No	Yes
Age							
	≤55	175/39	1.00	1.49 (0.88, 2.54)	325/67	1.00	2.81 (1.95, 4.05)
	>55	51/8	1.00	1.83 (1.05, 3.17)	175/49	1.00	2.02 (1.40, 2.93)
Sex							
	Female	124/24	1.00	1.27 (0.80, 2.00)	246/62	1.00	1.90 (1.40, 2.56)
	Male	102/23	1.00	1.35 (0.82, 2.21)	254/54	1.00	1.67 (1.19, 2.36)
BMI							
	<24	61/10	1.00	1.72 (0.84, 3.52)	149/27	1.00	2.06 (1.29, 3.30)
	≥24	165/37	1.00	1.19 (0.80, 1.760)	351/89	1.00	1.73 (1.32, 2.28)
Smoking							
	Never	143/30	1.00	1.21 (0.77, 1.91)	280/76	1.00	2.21 (1.63, 3.02)
	Ever/current	83/17	1.00	1.42 (0.81, 2.50)	220/40	1.00	1.41 (0.97, 2.07)
Drinking							
	Never	156/32	1.00	1.30 (0.85, 2.00)	298/75	1.00	1.76 (1.31, 2.37)
	Ever/current	70/15	1.00	1.34 (0.73, 3.44)	202/41	1.00	1.98 (1.34, 2.93)
Sleep hours							
	<7	87/20	1.00	1.40 (0.73, 2.69)	183/39	1.00	2.36 (1.47, 3.77)
	≥7	139/27	1.00	1.15 (0.74, 1.79)	317/77	1.00	1.55 (1.16, 2.08)
Diet score							
	≤2	87/20	1.00	1.55 (0.79, 3.03)	183/39	1.00	2.83 (1.83, 4.37)
	>2	139/27	1.00	1.02 (0.65, 1.59)	317/77	1.00	1.39 (1.02, 1.89)
Regular exercise							
	Unregular	87/20	1.00	1.25 (0.84, 1.88)	183/39	1.00	2.10 (1.60, 2.75)
	Regular	139/27	1.00	0.84 (0.39, 1.98)	317/77	1.00	1.13 (0.63, 2.01)

Models were adjusted for age (continuous), sex (female, male), BMI (continuous), smoking status (never smoked, current smoker), alcohol consumption (never drank, current drinker), systolic blood pressure (continuous), sleep duration (continuous), dietary factors (diet score ≤ 2, diet score >2), and regular exercise (regular, unregular).

**Table 4:** Sensitivity analysis for the association between parental diabetes and risk of offspring type 2 diabetes.

Variables		N/Cases	Model 3		Model 4		Model 5		Model 6	
			HR (95% CI)	*p*	HR (95% CI)	*p*	HR (95% CI)	*p*	HR (95% CI)	*p*
Paternal diabetes										
	No	4282/761	1.00		1.00		1.00		1.00	
	Yes	226/47	1.27 (0.88, 1.84)	2.10 × 10^−1^	1.30 (0.91, 1.87)	1.50 × 10^−1^	0.91 (0.60, 1.39)	6.70 × 10^−1^	0.89 (0.59, 1.37)	6.20 × 10^−1^
Maternal diabetes										
	No	3508/692	1.00		1.00		1.00		1.00	
	Yes	500/116	1.89 (1.47, 2.43)	7.00 × 10^−7^	1.90 (1.48, 2.43)	3.80 × 10^−7^	1.63 (1.24, 2.15)	5.00 × 10^−4^	1.63 (1.24, 2.14)	5.30 × 10^−4^
Combined										
	No parents	3845/661	1.00		1.00		1.00		1.00	
	Only maternal	437/100	1.95 (1.50, 2.55)	8.80 × 10^−7^	1.96 (1.51, 2.55)	5.20 × 10^−7^	1.69 (1.26, 2.25)	4.00 × 10^−4^	1.68 (1.26, 2.24)	4.30 × 10^−4^
	Only paternal	163/31	1.39 (0.89, 2.17)	1.50 × 10^−1^	1.42 (0.92, 2.20)	1.20 × 10^−1^	1.02 (0.61, 1.68)	9.60 × 10^−1^	1.00 (0.60, 1.66)	9.90 × 10^−1^
	Both parents	63/16	2.07 (1.12, 3.83)	2.10 × 10^−2^	2.15 (1.18, 3.92)	1.30 × 10^−2^	1.26 (0.61, 2.56)	5.60 × 10^−1^	1.23 (0.60, 2.51)	5.80 × 10^−1^

Model 3 adjusted for age (continuous), sex (female, male), BMI (continuous), smoking status (never smoked, current smoker), alcohol consumption (never drank, current drinker), systolic blood pressure (continuous), sleep duration (continuous), dietary factors (diet score ≤ 2, diet score >2) and regular exercise (regular, unregular). Model 4 further adjusted for fasting blood glucose (continuous) based on Model 3, Model 5 additionally adjusted for use of drugs (antihypertensive drugs, antidiabetic drugs, lipid-lowering drugs) based on Model 4. Model 6 additionally adjusted for history of cardiovascular disease (stroke, hypertension).

## Data Availability

The data presented in this study are available on request from the corresponding author. The data are not publicly available due to privacy.
